# Differential effects of synchronous and asynchronous multifinger coactivation on human tactile performance

**DOI:** 10.1186/1471-2202-8-58

**Published:** 2007-07-30

**Authors:** Tobias Kalisch, Martin Tegenthoff, Hubert R Dinse

**Affiliations:** 1 Department of Theoretical Biology, Institute for Neuroinformatics, Ruhr-University Bochum, D-44780 Bochum Germany; 2 Department of Neurology, BG-Kliniken Bergmannsheil, Ruhr-University Bochum, D-44789 Bochum Germany

## Abstract

**Background:**

Repeated execution of a tactile task enhances task performance. In the present study we sought to improve tactile performance with unattended activation-based learning processes (i.e., focused stimulation of dermal receptors evoking neural coactivation (CA)). Previous studies show that the application of CA to a single finger reduced the stationary two-point discrimination threshold and significantly increased tactile acuity. These changes were accompanied by an expansion of the cortical finger representation in primary somatosensory cortex (SI). Here we investigated the effect of different types of multifinger CA on the tactile performance of each finger of the right hand.

**Results:**

Synchronous and asynchronous CA was applied to all fingers of a subject's dominant hand. We evaluated changes in absolute touch thresholds, static two-point discrimination thresholds, and mislocalization of tactile stimuli to the fingertips. After synchronous CA, tactile acuity improved (i.e., discrimination thresholds decreased) and the frequency of mislocalization of tactile stimuli changed from directly neighboring fingers to more distant fingers. On the other hand, asynchronous CA did not significant improve tactile acuity. In fact, there was evidence of impaired tactile acuity. Multifinger CA with synchronous or asynchronous stimulation did not significantly alter absolute touch thresholds.

**Conclusion:**

Our results demonstrate that it is possible to extend tactile CA to all fingers of a hand. The observed changes in mislocalization of tactile stimuli after synchronous CA indicate changes in the topography of the cortical hand representation. Although single-finger CA has been shown to improve tactile acuity, asynchronous CA of all fingers of the hand had the opposite effect, suggesting the need for synchrony in multifinger CA for improving tactile acuity.

## Background

It is known that perceptual skills are permanently modified by the extent of use [1]. Intensive training enhances perceptual performance, and reduced use may impair specific perceptual abilities [2]. A large body of evidence indicates that training-induced improvement of somatosensory perception is associated with plastic changes in the cortical representations of the stimulated body part [3-8]. Generally speaking, the size of a cortical representation is positively correlated with the quality of performance of a specific task. The opposite effect has been shown for extreme forms of disuse, including immobilization or amputation of body parts, which lead to shrinkage or disintegration of cortical representations [9,10].

Over the past several years, we have attempted to establish training methods to improve human tactile perception on a several hours' time course using unattended, activation-based learning protocols. We developed a tactile stimulation protocol that enforces localized activation patterns in the brain and changes the functionality of cortical networks, resulting in enhanced tactile perception [11-17]. By costimulating a large number of mechanoreceptors in the fingertips, we provoked CA of cortical neurons in the related cortical finger representations, which resulted in a transient improvement of tactile acuity that could be demonstrated with a two-point discrimination task. The CA did not require subjects' attention or active participation. Therefore, it is considered to be a passive training method. The improvement in tactile acuity after 3 hours of tactile stimulation is considered to reflect Hebbian learning [18,19], whereby synchronous neural activity, which is believed to be fundamental to plastic changes, is provided by simultaneous tactile stimulation. To demonstrate the Hebbian nature of our CA protocol, we compared the effects of CA with single-site stimulation, in which only a single point of skin on the fingertips was stimulated. Although stimulation frequency and duration were the same in both paradigms, the single-site stimulation did not change cortical activation patterns or tactile acuity, clearly demonstrating the necessity for simultaneous activation of a large number of mechanoreceptors [13].

Previously, we investigated the cortical effects of CA, using non-invasive techniques, such as somatosensory-evoked potential mapping [12,16] and functional magnetic resonance imaging [13]. Both techniques allowed us to track CA-induced changes in the characteristics of the human finger representations in the primary somatosensory cortex (SI) in a pre/post paradigm. The results consistently revealed a selective increase in representation size of the coactivated fingers. As shown in a number of human and animal studies, there is usually a direct association between the extent of plastic changes at the cortical level and changes in behavioral performance [2,3,8,19]. In earlier experiments, we combined electroencephalogram and functional magnetic imaging data and data obtained from psychophysical tests of tactile acuity. We found a significant association between CA-induced cortical map changes and improved two-point discrimination [12,13,15]. Accordingly, subjects with a large degree of cortical reorganization showed a strong reduction in two-point discrimination thresholds (i.e., improved tactile acuity), while subjects that improved only little perceptually also showed small cortical reorganization.

CA is a passive tactile stimulation unrelated to any specific tactile task, by which a large number of receptors in the skin (relative to the density of their distribution, presumably Meissner cells und Merkel corpuscles [20,21]) are activated, which in turn remodels processing of tactile information in the somatosensory system. Therefore, it is conceivable that modification of perceptual performance is not limited to spatial two-point discrimination [18].

The present study describes experiments were we extended our attempts to modify tactile perception into two different directions. First, in order to investigate the conditions under which changes in tactile performance can be driven by stimulating not only a single finger, but all fingers of the dominant hand, we introduced a so-called multi-finger CA, in which all fingers of the dominant hand were stimulated. In that case, two options emerge: multifinger-CA can be done in a way, where all fingers are simultaneously stimulated, or asynchronously, i.e. in an uncorrelated way [22]. Previous experiments using small probes that allowed for stimulation of small areas on the tip of the index-finger had revealed that synchronous CA led to an improvement of tactile acuity, while uncorrelated stimulation impaired performance [18,23,24]. Second, in order to test the above described hypothesis, namely that CA due to its task-free nature modifies not only tactile acuity, we additionally to the assessment of 2-point thresholds measured absolute touch threshold and finger mislocalization performance.

## Results

All experiments were conducted in three subject groups that included 1) synchronous multifinger CA, 2) asynchronous multifinger CA, and 3) sham CA. Results are presented according to this group order. The influence of the different paradigms was analyzed in terms of absolute touch thresholds, two-point discrimination thresholds, and distribution of tactile stimuli mislocalization. For the sake of clarity, some acuity task thresholds were calculated as average values for the left and right hands. Nevertheless, statistical analyses were performed for single fingers.

### Effects of multifinger-CA on absolute touch thresholds

#### Synchronous multifinger CA

**Figure 1:**
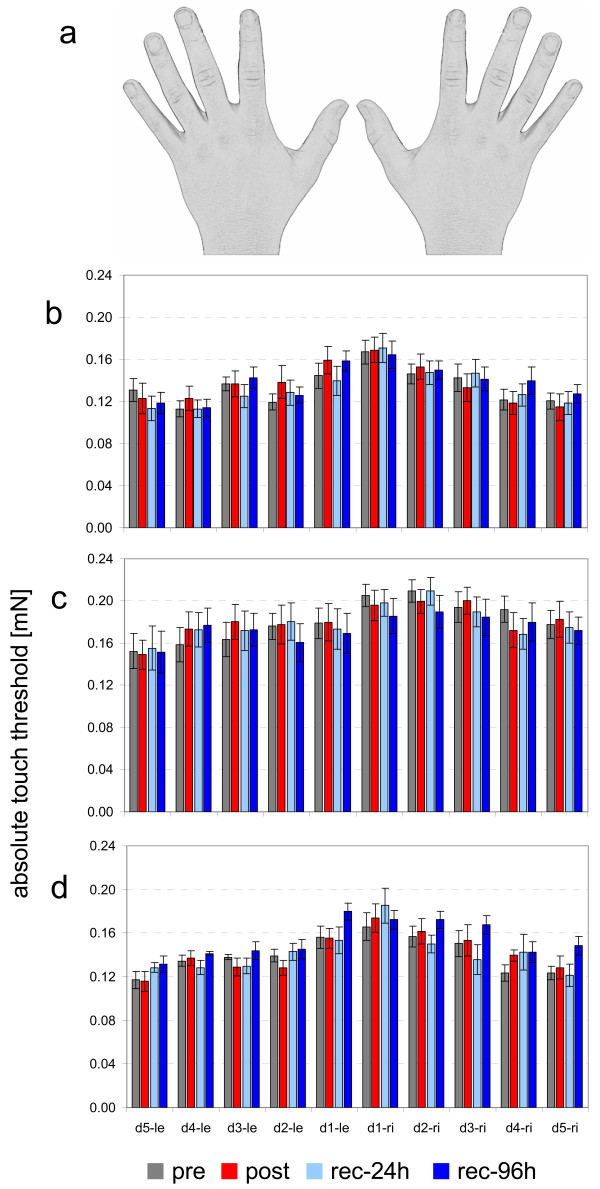
**Coactivation (CA) does not alter the absolute touch threshold**. (**a**) Sketch of the data arrangement in the depicted bar charts. Thresholds are arranged according to the position of the fingers. (**b**) Bar chart of the absolute touch thresholds of all fingers of the right and left hands before (pre) and at different time points (post, rec-24 h, rec-96 h) after synchronous CA. (**c**) Bar chart of the absolute touch thresholds of all fingers of the right and left hands before (pre) and at different time points (post, rec-24 h, rec-96 h) after asynchronous CA. (**d**) Bar chart of the absolute touch thresholds of all fingers of the right and left hands before (pre) and at different time points (post, rec-24 h, rec-96 h) after sham CA.

The average absolute touch threshold across all fingers of the right hand for subjects in the first group was 0.13 ± 0.03 mN in the pre session. This value changed to 0.14 ± 0.04 mN in the post session, 0.13 ± 0.03 mN in the rec-24 h session, and 0.14 ± 0.03 mN in the rec-96 h session. Threshold changes were investigated for each finger individually (repeated measures ANOVA for the factor SESSION), but no significant changes were observed (F_(3,39) _≤ 1.287, p ≥ 0.299). The sensory thresholds of the fingers decreased from the thumb to the little finger (Pearson correlation, r ≥ -0.964, p ≤ 0.008 for all sessions for the right hand; r ≥ -0.933, p ≤ 0.021 for post- and rec-24 h sessions for the left hand; Fig. 1b).

#### Asynchronous multifinger CA

The average absolute touch threshold across all fingers of the right hand for subjects in the second group was 0.18 ± 0.04 mN in the pre session. Thresholds for the post- and rec-24 h sessions were 0.18 ± 0.05 mN. In the rec-96 h session, the threshold changed to 0.17 ± 0.05 mN. As was the case with the first group, no significant changes were observed for single finger thresholds (repeated measures ANOVA for the factor SESSION, F_(3,39) _≤ 1.889, p ≥ 0.155). These results partially confirmed that there is a significant decrease in thresholds from the thumb to the little finger on each hand. That is, threshold decreases across fingers were significant in the pre session for the right (Pearson-correlation, r = -0.923, p = 0.026) and left (r = -0.983, p = 0.003) hands. The threshold changes were not significant for the fingers on the right or left hands in the post-, rec-24 h-, and rec-96 h sessions (r ≤ -0.865, p ≥ 0.058; Fig. 1c).

#### Sham CA

Absolute touch thresholds were also evaluated in the third group (i.e., subjects who did not receive CA). In the pre- and post sessions, the mean touch thresholds were 0.14 ± 0.02 mN. In the rec-24 h- and rec-96 h sessions, the mean touch thresholds were 0.14 ± 0.03 mN and 0.15 ± 0.02 mN. All means calculated for the right hand showed a decrease of absolute touch thresholds from the thumb to the little finger (Pearson correlation, r ≥ -0.867, p ≤ 0.048). This was the case for the left hand in the pre- and rec-24 h sessions (r ≥ -0.912, p ≤ 0.031), but not in the post- and rec-96 h sessions (r ≥ -0.867, p ≤ 0.057). As in the first and second groups, no significant changes were observed in the single finger thresholds (repeated measures ANOVA for the factor SESSION, F_(3,23) _≤ 3.246, p ≥ 0.052; Fig. 1d).

Summarizing the results of the first experiment, neither asynchronous nor synchronous CA altered subjects' ability to perceive light tactile stimulation (i.e., the absolute touch threshold). To avoid errors due to calculating absolute thresholds, we also evaluated threshold-difference percentages for each finger on the right and left hands with repeated measures ANOVA for the factors SESSION and DIFFERENCE. Analyses revealed no significant differences in pre- and post session data (F_(3,25) _≥ 0.526, p ≤ 0.592), pre- and rec-24 h data (F_(3,25) _≥ 0.457, p ≤ 0.634), or pre-and rec-96 h data (F_(3,25) _≥ 0.759, p ≤ 0.121) for synchronous multifinger CA, asynchronous multifinger CA, and sham CA groups.

### Effects of multifinger CA on two-point discrimination

As mentioned in the methods section, all subjects attended a training session prior to participating in the first (i.e., pre) session of the two-point discrimination task. Because the training session was the same for all three groups, the data from the training and pre sessions could be compared to evaluate the test-retest reliability of the two-point discrimination paradigm. The average discrimination threshold for all subjects was 1.62 ± 0.23 mm in the training session and 1.63 ± 0.26 mm in the pre session (t-test, p = 0.345). Comparison of the mean thresholds for each session revealed a high test-retest reliability (Cronbach's α = 0.882).

The single-needle condition of the two-point discrimination apparatus was used as a control condition. The average hit rate in the control condition was 0.79 ± 0.08 in the training session and 0.79 ± 0.07 in the pre session (t-test, p = 0.385). (For further information about the two-point discrimination paradigm and Signal Detection Theory see ref. [12]).

#### Synchronous multifinger CA

**Figure 2:**
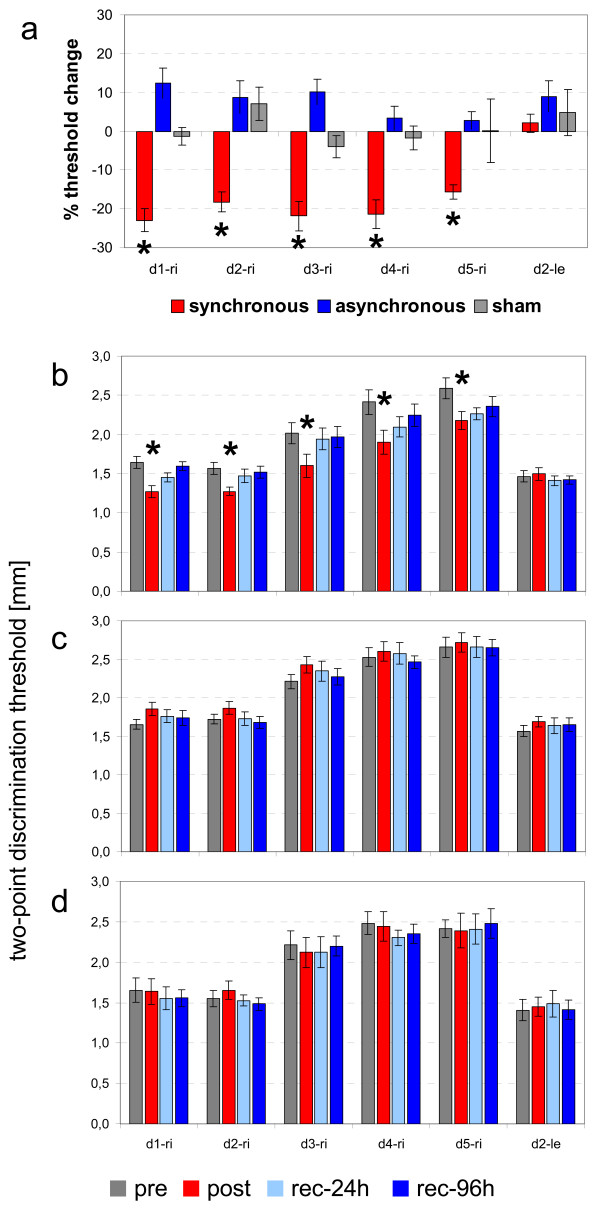
**Synchronous coactivation (CA) improves two-point discrimination**. (**a**) Bar chart of the percentage changes in two-point discrimination thresholds after synchronous, asynchronous, and sham CA. (**b**) Bar chart of two-point discrimination thresholds of all fingers of the right hand (d1-ri – d5-ri) after synchronous CA and the left non-stimulated index finger (d2-le). (**c**) Bar chart of two-point discrimination thresholds of all fingers of the right hand (d1-ri – d5-ri) after asynchronous CA and the left non-stimulated index finger (d2-le). (**d**) Bar chart of two-point discrimination thresholds of all fingers of the right hand (d1-ri – d5-ri) after sham CA and the left non-stimulated index finger (d2-le). * Significant change (p ≤ 0.05)

Prior to CA, the average discrimination threshold was 2.05 ± 0.37 mm for all fingers on the right hand and 1.47 ± 0.23 mm for the left index finger. Repeated measures ANOVA for the factor SESSION revealed a significant change in threshold for each finger on the right hand in the four sessions (F_(3,39) _≥ 11.930, p ≤ 0.001), but not for the left, non-coactivated index finger (F_(3,39) _= 1.130, p = 0.355; Fig. 2b). Post-hoc analysis with an LSD test revealed a consistent, significant threshold change from pre- to post sessions for the thumb (1.65 ± 0.08 mm to 1.27 ± 0.24 mm, p = 0.010), index finger (1.57 ± 0.24 mm to 1.27 ± 0.17 mm, p = 0.007), middle finger (2.02 ± 0.43 mm to 1.60 ± 0.47 mm, p = 0.043), ring finger (2.41 ± 0.50 mm to 1.90 ± 0.49 mm, p = 0.018), and little finger (2.59 ± 0.43 mm to 2.18 ± 0.36 mm, p = 0,018). Although threshold changes were detectable only within 24 h of the experiment, there appeared to be a prolonged effect of multifinger CA (average rec-96 h threshold, 1.94 ± 0.34 mm; post-hoc test, p ≥ 0.087). Initial discrimination thresholds showed a typical distribution (i.e., an increase from the thumb to the little finger; Pearson correlation, r = 0.954; p = 0.012; Fig. 2b).

#### Asynchronous multifinger CA

The initial discrimination threshold was 2.06 ± 0.28 mm for all fingers on the right hand and 1.57 ± 0.24 mm for the left index finger (Fig. 2c). Repeated measures ANOVA for the factor SESSION revealed significant threshold changes for the thumb and middle finger on the right hand (F_(3,39) _≥ 3.046, p ≤ 0.046), whereas no significant changes were observed for the right index finger (F_(3,39) _= 2.734, p = 0.063), ring finger (F_(3,39) _= 1.146, p = 0.349), and little finger (F_(3,39) _= 0.616, p = 0.611) or the left index finger (F_(3,39) _= 1.353, p = 0.278). Post-hoc analyses of the threshold differences for the right thumb and middle finger with an LSD test gave results that contradicted the repeated measures ANOVA. For instance, differences between single session thresholds were not significant for the thumb (p ≥ 0.098) or the middle finger (p ≥ 0.168).

#### Sham CA

The two-point discrimination task revealed an average discrimination threshold of 2.06 ± 0.33 mm during the pre session on all fingers of the right hand and 1.41 ± 0.33 mm for the left index finger. Repeated measures ANOVA for the factor SESSION conducted for each finger yielded no significant changes in discrimination thresholds for any fingers on the right hand (F_(3,23) _≤ 5.711, p ≥ 0.107) or the left index finger (F_(3,23) _= 0.310, p = 0.818; Fig. 2d).

The results of the second experiment demonstrated that synchronous multifinger CA reliably elicited changes in tactile acuity (i.e., two-point discrimination abilities; Fig. 2a). Additionally, there was a trend towards a cumulative effect of multifinger CA on the duration of the evoked changes, because unlike the situation with single-finger CA, there was no full recovery after 24 h (Fig. 2b). On the other hand, after asynchronous CA, there was a tendency toward increased two-point discrimination thresholds for some fingers, but the significance criteria were not met, and the results were comparable to those of the sham CA group, in which no changes in tactile acuity were observed (Fig. 2a).

### Effects of multifinger CA on mislocalization behavior

#### Synchronous multifinger CA

**Figure 3:**
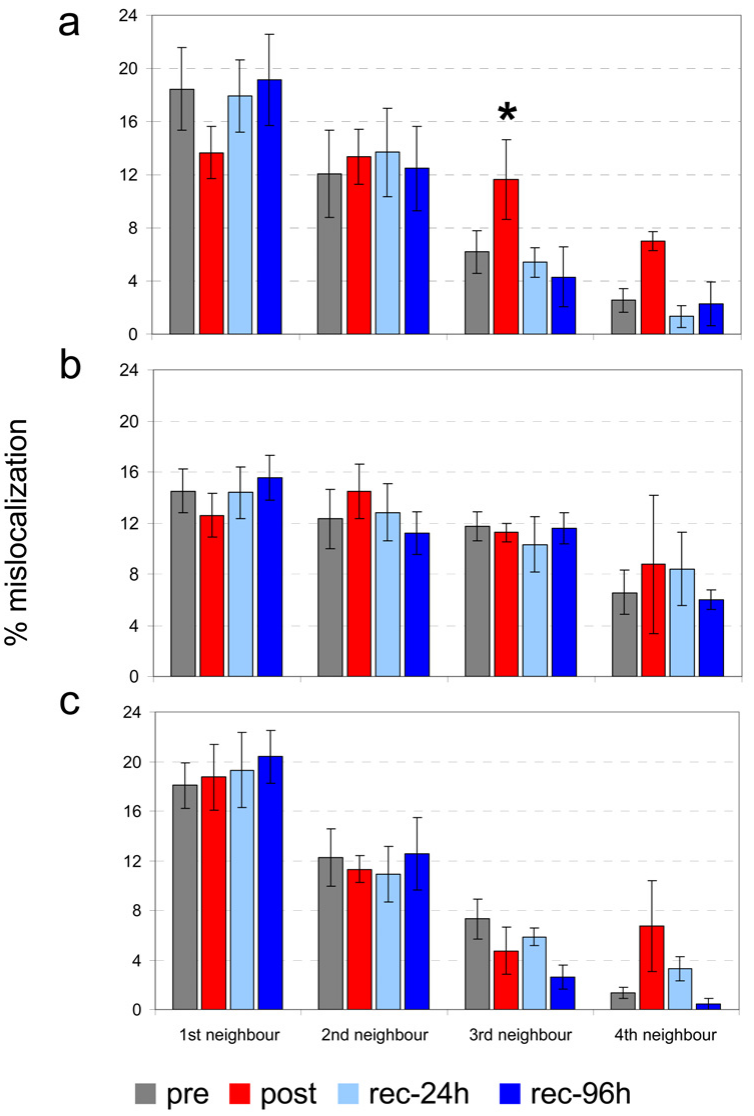
**Synchronous coactivation (CA) changes in mislocalization behavior**. (**a**) Percentage changes in rate of mislocalization to fingers other than the stimulated one before (pre) and at different time points (post, rec-24 h, rec-96 h) after synchronous CA. (**b**) Percentage changes in rate of mislocalization to fingers other than the stimulated one before (pre) and at different time points (post, rec-24 h, rec-96 h) after asynchronous CA. (**c**) Percentage changes in rate of mislocalization to fingers other than the stimulated one before (pre) and at different time points after (post, rec-24 h, rec-96 h) sham CA. * Significant change (p ≤ 0.05)

Repeated measures ANOVA for the factor SESSION was used to evaluate changes in the number of stimulus mislocalizations to the first (F_(3,9) _= 1.329, p = 0.292), second (F_(3,39) _= 0.351, p = 0.789), third (F_(3,39) _= 6.960, p = 0.010), and fourth (F_(3,9) _= 4.282, p = 0.132) neighboring digits. A significant change in the number of mislocalizations to the third neighboring digit was observed, and the result was further analyzed with an LSD post-hoc test. We found that the frequency of mislocalization to the third neighboring digit was significantly increased in the post session (p ≤ 0.031; Fig. 3a). Additionally, there were differences in the number of mislocalizations to the first and fourth neighboring digits in the post session, but they were not significant (p ≤ 0.093).

#### Asynchronous multifinger CA

Mislocalization behavior was investigated on all coactivated fingers of the right hand. Across the four sessions there was no significant change in the frequency of mislocalization or mislocalization to the first (repeated measures ANOVA, F_(3,39) _= 0.831, p = 0.492), second (F_(3,39) _= 1.008, p = 0.416), third (F_(3,39) _= 0.134, p = 0.738), or fourth (F_(3,39) _= 0.463, p = 0.728;Fig. 3b) neighboring digits.

#### Sham CA

There were significant changes in the frequency of mislocalizations to fingers other than the stimulated finger in the sham condition. This held for mislocalizations to the first (F_(3,23) _= 0.430, p = 0.733), second (F_(3,23) _= 0.738, p = 0.545), third (F_(3,23) _= 3.050, p = 0.085), and fourth (F_(3,23) _= 2.264, p = 0.260) neighboring digits (Fig. 3c).

To summarize the third experiment, there were differences in mislocalization after synchronous CA but not after asynchronous and sham CA. After synchronous CA there was a tendency for mislocalization to shift from neighboring digits to distant digits.

## Discussion

The application of synchronous and asynchronous multifinger CA resulted in differential effects on tactile perception. Behavioral changes, such as improved two-point discrimination and changes in mislocalization frequency were observed after synchronous multifinger CA. In contrast, there was a trend towards impaired two-point discrimination performance after asynchronous multifinger CA. This was surprising because the stimulation protocol that was used for multifinger CA was previously shown to effectively improve tactile acuity (i.e., two-point discrimination performance) on a single stimulated finger [13-15]. Thus, there appears to be a necessity for synchronous stimulation not only in the range of a single finger but also between all fingers of a hand-when multifinger CA is applied.

### Connections of functional and behavioral changes evoked by peripheral stimulation

In animal experiments [7,22], synchronous stimulation evoked "melting" of originally separate cortical representations, whereas asynchronous stimulation caused segregation of the cortical representations [23]. Cortical reorganization in animals and behavioral changes in humans were maximal when stimulation was absolutely synchronous and gradually diminished with increasing delay between the two stimulus trains [24]. Braun and colleagues [25,26] used electroencephalogram and magnetoencephalography recordings to investigate associations of tactile discrimination training, cortical organization, and mislocalization behavior. They reported an increase in the size of the cortical representations of the fingers involved in the tactile training. Consequently, there was overlap of the neural networks that formed the digit representations. Furthermore, the authors reported localization errors of near-threshold tactile stimuli to fingers that where included in the stimulation procedure.

Further evidence for a functional interaction of training, tactile acuity, and mislocalization of tactile stimuli comes from Pilz and coworkers [27], who investigated the effects of synchronous and asynchronous stimulation on human finger representations by means of tactile CA. They demonstrated that synchronous stimulation leads to an overlap of finger representations in SI and an increase in the frequency of mislocalization. On the other hand, asynchronous stimulation resulted in a merging of cortical representations and a decrease in the number of mislocalizations between the stimulated fingers.

In the present study, we show that synchronous multifinger CA of all fingers on a hand leads to improved tactile acuity (i.e., diminished two-point discrimination thresholds) and changes in typical mislocalization behavior (i.e., decreasing numbers of mislocalizations with increasing distance from the stimulated finger).

Associations between perceptual discrimination and cortical organization in the human somatosensory system have also been studied in blind subjects. Sterr and co-workers [28,29] showed that blind multifinger Braille readers, who have expert tactile acuity due to daily training in Braille, had an increased frequency of mislocalization between the fingers of the hand used for Braille reading. The reading fingers were less prone to mislocalization of near-threshold stimuli, although tactile stimulation of most of the other fingers of the hand was commonly mislocalized to the reading fingers. Mislocalizations occurred more frequently on the fingers of the reading hand. Thus, it was assumed that increased mislocalization was somehow related to Braille reading skills rather than blindness in general. In all of the experiments mentioned, the observed changes in mislocalization could be due to an anchorage effect of attention rather than reorganization of somatosensory cortex. The anchorage effect describes a sensory perception whereby stimulus tends to be localized to certain points of reference on the skin [30-32]. Based on this hypothesis, one can assume that passive stimulation or active training of some fingers attracts attention to those fingers and creates anchor points that influence mislocalization behavior. In the present study, we included all right hand fingers in multifinger CA, which controlled for differences in attention to particular.

### Synchronous multifinger CA is capable of improving tactile acuity

Multifinger CA mimics the training-related perceptual performance demonstrated in Braille readers [28] and experienced sighted controls [26] on a short time scale. The perception of mechanical stimuli (i.e., the absolute touch threshold), which is known to be improved in blind Braille readers [33], remained unaffected after multifinger CA, regardless of whether synchronous or asynchronous stimulation was applied. This indicates that the absolute touch threshold cannot be altered by the stimulation we used. This result is consistent with our previous study [18] showing that CA protocols of different length and frequency, applied to a single finger, do not change absolute touch perception. The differential effects of specific peripheral stimulation protocols became obvious when it was demonstrated that the absolute touch threshold could be changed by electrical transmission of white noise to the dermal receptors. It was assumed, therefore, that a stochastic resonance mechanism elicited changes in touch perception [34].

Recent research on plastic changes in the somatosensory system provides explanations for changes evoked by simultaneous input in terms of long-term active training [1,2,19,35] and short-term stimulation [13,14,18]. Our own studies provided evidence for enlarged finger representations in SI following single-finger CA. These changes indicate functional reorganization of the respective neural networks. Based on the results mentioned above, one can assume that synchronous multifinger CA leads to an enlargement of all finger representations and, therefore, increases overlap of corresponding neural fields. Strengthening connections within a single representation may lead to improved discrimination performance, as shown with the two-point discrimination task. Overlap of neighboring and more distant finger representations may alter localization behavior, and consequently, cause mislocalization to more distant fingers.

Previous CA studies suggest that highly synchronous tactile stimulation changes the neural networks that underlie representations in primary sensorimotor cortices in a Hebbian manner. To test that hypothesis, single-finger CA was applied with a device delivering a single point-like stimulus rather than widespread stimuli to a fingertip [12]. Although stimulation frequency and duration were the same as in the single-finger CA, no changes of tactile acuity or cortical activation were initiated by this kind of stimulation. This demonstrates the need for broad synchronous activation across a number of receptive fields to alter functional mechanisms in the sensory system. The present study extends that finding and demonstrates for the first time that there is a need for synchronous stimulation when CA is applied to all fingers of a hand. Improvement of single finger tactile performance is completely suppressed when asynchronous stimulation is applied to the fingers. The suppressive influence of asynchronous stimulation of separate fingers seems to have a wide-spread effect on cortical level. Otherwise, the effects of lateral inhibition would have become obvious (i.e., a weaker suppression of tactile performance) in distal fingers, such as the thumb and little finger. The absence of systematic threshold changes between asynchronously stimulated fingers supports this view.

This result of CA applied across fingers is somewhat surprising given that CA on the scale of the fingertip has already positive effects on tactile acuity, when delivered in a synchronous way. Only when applied in an uncorrelated, asynchronous mode, tactile acuity became impaired [23,24]. Our data suggest that the improving effect of simultaneous CA applied to a single fingertip is overridden in a condition, in which the tips of each finger is synchronously activated, but in combination with an asynchronously activation applied to each finger.

In addition to examining the effects of tactile multifinger CA, we were able to evaluate tactile acuity based on two-point discrimination performance and absolute touch perception. The two-point discrimination task is discussed controversially in the literature [36,37], however we have used it rather than the grating orientation task with great success during the last years [38]. Our primary interest was in the relative changes in discrimination thresholds after activation-based learning (i.e., CA) rather than absolute discrimination thresholds. As we show in the present study, the test-retest reliability of the discrimination paradigm is very high therefore allowing to investigate reliably CA-related changes in tactile acuity. According to unpublished results from our group, acuity thresholds obtained with gratings and two-point discriminations are very similar yielding a high linear correlation, although thresholds obtained with gratings are slightly lower in general.

### Relation of absolute touch and two-point discrimination thresholds

It is known that tactile acuity of the fingertips is not distributed equally, but is best at the thumb and index finger and declines across the remaining fingers [39-41]. In the present study, we demonstrated that two-point discrimination abilities and touch perception abilities are complementry. For instance, absolute touch thresholds decline from thumb to little finger, whereas two-point discrimination thresholds increase in that direction. This seeming contradiction may be explained by marginal differences in the skin structure of the fingers, which can be compensated for in the two-point discrimination task by self-adjusting the contact intensity between the skin and the testing device, but which become obvious when perceiving light mechanical stimuli. The finding that the thumb is less sensitive than other fingers and that fingers on the left hand are more sensitive than fingers on the right hand are in line with of the results of Sterr and colleagues [28], who demonstrated this effect in blind subjects and sighted controls. Slight differences in absolute touch thresholds for mechanical stimuli may be attributed to use-dependent changes in skin structures. It can be assumed that the skin of the thumb is exposed to higher mechanical loads than the other fingers because of the opposing arrangement of the remaining fingers on the hand. Furthermore the entire right hand is required in nearly all unilateral processes in right-handed persons, which may explain contralateral differences in skin structure and discrepancies in touch perception.

## Conclusion

We demonstrated that synchronous and asynchronous multifinger CA evoke differential effects in the human somatosensory system and differentially affect perceptual abilities. Although CA has been shown to improve tactile acuity of a single finger [11,13,14,42], no effect was observed when asynchronous CA was applied to all of the fingers on a hand. In contrast, synchronous multifinger CA improved tactile acuity and demonstrated the need for synchronicity in peripheral stimulation protocols. Consequently, synchronous multifinger CA can be regarded as a step forward in transforming activation-based, unattended learning protocols to more applicable procedures for rehabilitation and restoration of basic tactile abilities.

## Methods

### Two-point discrimination test

Tactile two-point discrimination was accomplished using the constant stimuli method described previously [12,14,15]. Rather than using hand-held probes, we used a specially designed apparatus that could be applied at a fixed position on the skin of the fingertips for approximately 1 s and allowed rapid switching between test conditions (i.e., 7 needle distances ranging from 0.7 to 2.5 mm and a single needle to test for false alarms). Subjects were instructed to 1) place the finger on the support without force 2) maintain the initial position of the finger respectively refrain from making exploratory movements with the fingertip and 3) classify all stimuli they could not clearly discriminate as "1" and stimuli that could be clearly discriminated as "2,".

The brass needles we used for stimulation were 1.9 mm in length. They were 0.7 mm in diameter with blunt ends that were approximately 200 μm in diameter. Because the application force corresponded to the tare weight of the tested finger, it was assumed to be ~100–150 g. These physical boundary conditions ensured punctual application of the tactile stimuli and avoided any pain sensation during testing. During testing, all eight test conditions were presented eight times for a total of 64 tests per session. The subjects were not informed of the ratio of needle pairs to single needles (i.e., 7:1). After placing their fingertips in the test position by lowering the arm support, subjects had to decide immediately if they had the sensation of one or two needles (i.e., two-alternative forced choice test). The sums of their responses were plotted against the needle distances, producing a psychometric function that was fitted by a binary logistic regression using SPSS software (SPSS Inc., Chicago, Illinois). Threshold was taken from the fit where 50% correct responses were reached.

All subjects attended a training session to become familiar with the testing procedures 15 minutes before formal testing began. Although significant differences between discrimination thresholds obtained by grating orientation discrimination tests and two-point discrimination tests were reported in the past [43], the test-retest reliability was comparable when the two-point discrimination was conducted in the described way (unpublished data).

### Absolute touch threshold

Fine-touch sensitivity was evaluated by probing the fingertips with von Frey filaments (Marstocknervtest, Marburg, Germany), following the procedures described with Semmes-Weinstein monofilaments [44]. Each filament was calibrated to a known buckling force determined by its length and diameter. The von Frey test kit contains 16 different filaments calibrated to forces ranging from 0.25–294 mN in logarithmic scaling. An additional two filaments with forces of 0.08 mN and 0.20 mN were used to expand the effective test range (Touch Test, Stoelting Co, Wood Dale, Illinois). Fine-touch sensitivity was tested with a staircase procedure, during which subjects were required to indicate whenever they perceived an indentation. The applied contact forces were decreased in a step-wise manner until the subjects no longer perceived the stimulus (lower boundary) and then increased until the stimulus was again perceived (upper boundary). This procedure was repeated three times, resulting in six values that were averaged to form the absolute touch threshold.

### Mislocalization test

Mislocalization of stimuli to fingers other than the stimulated finger has not been studied for some time since most studies make use of above-threshold stimulation, thereby avoiding mislocalizations [33]. On the other hand, mislocalizations were often excluded from analysis because they were presumed to be outliers [45]. Schweizer and colleagues [46,47] began to systematically investigate mislocalization behavior following tactile stimulation near the absolute touch threshold to the fingertips. They showed that localization errors obeyed a somatotopic principle whereby stimuli are preferentially mislocalized to sites adjacent to the stimulated skin region and differ significantly from guessing behavior. These results where confirmed by objective, apparatus-based, measurements of tactile mislocalizations [48]. In the present study, we used a set of von Frey monofilaments (0.25–294 mN) and touch test filaments (0.08 and 0.20 mN) to conduct a five-alternative forced choice detection test on the fingertips of the right and left hands (for a detailed description of the test, see ref. [46]). Each finger was stimulated 20 times in randomized order. Each correct response was followed by stimulation of lower intensity, and each false response was followed by stimulation of higher intensity to the same finger. Using this procedure, the error rate (i.e., the number of mislocalizations) was adjusted to ~50%.

Mislocalizations were analyzed according to their distribution on the fingers. In cases where the staircase procedure did not result in 50% mislocalizations, the data were normalized to 10 mislocalizations from each finger. To achieve an overview of individual mislocalization behavior, the determined extent of mislocalizations from each finger to any other finger was categorized as mislocalization from the stimulated finger to the first, second, third, or fourth neighboring finger. This categorization resulted in eight first, six second, four third, and two fourth neighbor fingers. Mislocalizations from the stimulated finger to the first, second, third, and fourth neighboring fingers were averaged for each hand of each subject in each session.

### Multifinger CA

The principle of multifinger CA is derived from the CA procedure as described in our previous studies [13,14,42]. Five small solenoid devices were fixed to the tips of all fingers on the right hand. Mechanoreceptors in the fingers were simultaneously stimulated (i.e., coactivated) with the solenoid devices. The CA stimuli (pulse duration, 10 ms; interstimulus interval, 100–3000 ms, according to a Poisson distribution; average frequency, 1 Hz) were played back via portable digital devices, permitting unrestrained subject mobility during CA.

For asynchronous multifinger CA, three digital devices were used. Because each of the devices was capable of repeating stimuli through two separate channels, it was possible to provide five absolute asynchronous stimulation sequences to the fingers of the right hand. For synchronous multifinger CA, we used a custom-made amplifier that was triggered by a digital device that transferred stimuli to five connected solenoids, thereby stimulating all fingers of a hand simultaneously. Synchronous and asynchronous multifinger CA was applied for 3 hours (for further information about the mechanisms of CA and results of previous experiments see refs. [13,14,18]).

### Participants and groups

The study was performed in accordance with the Declaration of Helsinki. All Subjects gave their written informed consent, and the protocol was approved by the local ethical committee of the Ruhr-University Bochum. A total of 26 subjects were recruited by advertisement from the university community to participate the presented study. All subjects were right-handed according to the Edinburgh Handedness Inventory [49]. Ten subjects received synchronous multifinger CA (3 males, 7 females; aged 23.2 ± 2.7 years), another 10 subjects received asynchronous multifinger CA (4 males, 6 females; aged 24.6 ± 2.7 years), and six subjects underwent sham CA (3 males, 3 females; aged 24.5 ± 0.6 years). Subjects were financially compensated for participation.

### Experimental schedule

All psychophysical experiments were carried out prior to multifinger CA (i.e., pre session) and three times after multifinger CA, in order to evaluate long-term changes in tactile performance and possible recovery of effects. One session was conducted immediately after multifinger CA (i.e., post session), another 24 hours later (i.e., rec-24 h), and the last one 4 days after multifinger CA (i.e., rec-96 h).

## Authors' contributions

TK conceived of the study, carried out the psychophysical measurements, performed the statistical analyses, and drafted the manuscript. MT participated in the study design and helped to draft the manuscript. HRD participated in the study design and coordination and helped to draft the manuscript. All authors read and approved the final manuscript.
